# Correlation analysis of Serum FGLP1, UACR and the risk of heart failure in patients with type 2 diabetes

**DOI:** 10.5937/jomb0-65295

**Published:** 2026-06-16

**Authors:** Lianheng Xia, Yingshuo Guo, Jiaxin Wang, Tingxuan Wang, Chunhui Xu, Meiyu Song, Chang Du, Qingjie Wang, Jie Gao

**Affiliations:** 1 First Affiliated Hospital of Heilongjiang University of Traditional Chinese Medicine, Vascular Surgery Department, Harbin, China; 2 Fushun Central Hospital, Cardiovascular Medicine Department, Fushun, China; 3 Armed Police Corps Hospital of Heilongjiang, Harbin, China; 4 The First Affiliated Hospital of Xiamen University, Endocrinology Department, Xiamen, China

**Keywords:** type 2 diabetes, left ventricular diastolic dysfunction, fibrinogen-like protein 1 C-terminal peptide, urinary albumin-to-creatinine ratio, dijabetes tipa 2, dijastolna disfunkcija leve komore, C-terminalni peptid proteina sličnog fibrinogenu 1, odnos albumin/kreatinin u urinu

## Abstract

**Background:**

To explore the correlations between serum FGLP1 (Fibrinogen-like protein 1 C-terminal peptide) levels, the urine albumin-to-creatinine ratio (UACR) and left ventricular diastolic dysfunction (LVDD) in elderly patients with type 2 diabetes mellitus (T2DM).

**Methods:**

From August 2023 to August 2025, 256 elderly patients with T2DM who were admitted to our hospital were selected as the case group, and 120 healthy individuals who underwent physical examinations were selected as the control group. In accordance with the diagnostic criteria for LVDD, T2DM patients were divided into a disorder group (n=70) and a nondisorder group (n = 186). Single-factor analysis, multivariate logistic regression, and Spearman correlation analyses were performed to examine the relationships among serum FGLP1 levels, UACRs, and LVDD in T2DM patients, and receiver operating characteristic curves were used to evaluate the ability of serum FGLP1 levels and urine UACRs to predict LVDD.

## Abstract (sr)

**Uvod:**

Cilj je bio da se ispita povezanost između nivoa serumskog FGLP1 (C-terminalnog peptida proteina sličnog fibrinogenu 1), odnosa albumin/kreatinin u urinu (UACR) i dijastolne disfunkcije leve komore (LVDD) kod starijih pacijenata sa dijabetesom melitus tipa 2 (T2DM).

**Metode:**

U periodu od avgusta 2023. do avgusta 2025. godine, 256 starijih pacijenata sa T2DM hospitalizovanih u našoj ustanovi uključeno je kao ispitivana grupa, dok je 120 zdravih ispitanika koji su obavili sistematski pregled činilo kontrolnu grupu. U skladu sa dijagnostičkim kriterijumima za LVDD, pacijenti sa T2DM su podeljeni na grupu sa poremećajem (n=70) i grupu bez poremećaja (n = 186). Radi ispitivanja povezanosti između nivoa serumskog FGLP1, UACR i LVDD kod pacijenata sa T2DM primenjene su univarijantna analiza, multivarijantna logistička regresija i Spirmanova korelaciona analiza, dok su ROC krive korišćene za procenu prediktivne vrednosti serumskog FGLP1 i UACR za LVDD.

**Rezultati:**

Koncentracija serumskog FGLP1 i UACR su bile više u ispitivanoj nego u kontrolnoj grupi (P&lt;0,05). Utvrđene su statistički značajne razlike u starosti, trajanju bolesti, koncentraciji serumskog FGLP1 i UACR između grupa sa i bez LVDD (P&lt;0,05).

**Zaključak:**

Nivoi serumskog FGLP1 i UACR u urinu kod starijih pacijenata sa dijabetesom melitus tipa 2 su usko povezani sa LVDD. Kombinovano određivanje ova dva pokazatelja može doprineti ranoj dijagnozi LVDD.

## Introduction

Type 2 diabetes (T2DM) is a common chronic metabolic disorder with a high incidence rate among elderly individuals [1]. Left ventricular diastolic dysfunction (LVDD) is a common consequence of type 2 diabetes that has a significant impact on patients' prognosis and quality of life. T2DM frequently coexists with cardiovascular illnesses [2]. Improving the cardiovascular outcomes of individuals with type 2 diabetes requires early detection and correction of LVDD [3]. Serum FGLP1 is a newly discovered adipokine that plays important roles in glucose metabolism and energy balance and is related to the occurrence and development of cardiovascular diseases [4].

With the continuous increase in the global incidence of diabetes, cardiovascular complications related to type 2 diabetes (T2DM) have become the main factor affecting the prognosis of patients. Among them, left ventricular diastolic dysfunction (LVDD), as an early manifestation of cardiac dysfunction, is crucial for preventing the occurrence of heart failure. In recent years, fibrinogen-like protein 1 C-terminal peptide (FGLP1), a newly discovered adipokine, and urine albumin/creatinine ratio (UACR), a sensitive indicator of renal injury, have attracted increasing attention in cardiovascular disease. However, the association between these two indicators and LVDD in T2DM patients, and their predictive value still needs to be further clarified. This study aimed to investigate the correlation between serum FGLP1 levels and UACR in elderly T2DM patients with LVDD, with the intention of providing new biomarker evidence for the early identification of diabetic cardiac complications and to have important clinical significance for improving the prognosis of T2DM patients.

The ratio of urinary albumin to creatinine (UACR) is an important indicator of early kidney damage. It is also associated with an increased risk of cardiovascular diseases [5]. The relationship between serum FGLP1 levels, urinary UACRs, and LVDD in older individuals with type 2 diabetic mellitus (T2DM) has not been extensively studied to date. The purpose of this study is to investigate the connections among the three to offer new concepts and goals for the early detection and management of cardiovascular disease in individuals with type 2 diabetes.

## Materials and methods

### General information

From August 2023 to August 2025, 256 elderly patients with T2DM who were admitted to our hospital were selected as the case group, and 120 healthy individuals who underwent physical examinations were selected as the control group.

Inclusion criteria: (1) aged ≥ 60 years; (2) met the diagnostic criteria for T2DM [6]; and (3) had no known abnormalities in blood sugar or lipid levels.

Exclusion criteria: (1) severe acute complications of diabetes or other severe cardiovascular diseases (such as coronary heart disease, cardiomyopathy, heart valve disease, etc.); (2) severe liver and kidney dysfunction, malignant tumours, or autoimmune diseases; and (3) current medication use that interferes with UACR or FGLP1. There were no statistically significant differences (P>0.05) between the two groups in age, sex, body mass index, or educational attainment (see Table 1).

**Table 1:** Comparison of general data between the two groups [x̄±s or n/n].

Group	n	Age <br>(years)	Gender <br>(Male/Female)	Body Mass Index <br>(kg/m^2^)	Cultural level (high school or <br>vocational school or below)/College or <br>undergraduate degree or above
Case group	256	75.53±8.36	130/126	21.39±1.45	154/102
Health Group	120	74.95±7.31	62/58	21.31 ±1.56	78/22
t/c^2^		0.464	0.016	0.606	0.409
P		0.648	0.913	0.540	0.527

This research received approval from the hospital's ethical committee (A0289), and patients or their families provided informed consent.

### Case collection

Clinical data from patients, including sex, age, height, systolic blood pressure (SBP), diastolic blood pressure (DBP), heart rate (HR), duration of diabetes, smoking history, and alcohol consumption history, were collected from the hospital's electronic medical record system.

### Laboratory indicators

All participants fasted for more than 8 hours before blood collection. After a morning fast, peripheral venous blood was drawn, and the serum was separated using centrifugation. Using an enzyme-linked immunosorbent test (ELISA) and a reagent kit acquired from ABC Company in the US, the amount of FGLP1 was measured. All directions were meticulously followed during the operation. The standard solution had to be calibrated before use to ensure test results were precise. The validity period, batch number, storage conditions, etc., of the reagents were checked to ensure that they met the requirements. Moreover, quality control samples were used to verify the reagents' performance and ensure the accuracy and reliability of the test results. Morning urine was collected, and UACR was determined by the immunoturbidimetric method. The instrument used was the Japanese Toshiba TBA-200FR automatic biochemical analyser. Moreover, indicators such as osteoprotegerin (OPG), soluble nuclear factor-kB receptor activator ligand (sRANKL), plasma creatinine (Cr), uric acid (UA), total cholesterol (TC), triglycerides (TG), low-density lipoprotein cholesterol (LDL-C), high-density lipoprotein cholesterol (HDL-C), urea nitrogen (BUN), and insulin-like growth factor-1 (IGF-1) were also detected.

### Echocardiography examination

An experienced echocardiologist, using the Philips IE33 colour Doppler ultrasound diagnostic instrument, performed transthoracic echocardiography on patients with T2DM. Heart structural markers were assessed, including interventricular septum thickness (IVS) and left ventricular end-diastolic diameter (LVEDD). The ratio of peak diastolic early-to-late mitral valve flow velocity (E/A) was determined by Doppler ultrasound. The left ventricular ejection fraction (LVEF) was determined using the modified two-plane Simpson method. The diagnostic criterion for LVEDD was an E/A<1. Based on echocardiographic results, patients with T2DM were divided into the LVDD and non-LVDD groups [7].

### Statistical analysis

Data analysis was conducted using SPSS 25.0. The c^2^ test or Fisher's exact probability method was used to compare groups. Count statistics are expressed as the number of instances and percentages. The Kolmogorov-Smirnov test was used to determine whether the measurement data were normally distributed. Normally distributed data are expressed as x̄±s, and comparisons between groups were performed using two independent sample t tests; normally distributed data are expressed as M (P25, P75), and comparisons between groups were performed using the rank sum test. Multivariate logistic regression was used to identify independent risk factors for LVDD in elderly patients with T2DM. Spearman correlation analysis was used to investigate relationships among LVDD, serum FGLP1 levels, and UACR. Receiver operating characteristic (ROC) curves were constructed to evaluate the ability of serum FGLP1 levels and urine UACR to predict Lv DD.

## Results

### Comparison of serum FGLP1 levels and urine UACR between the two groups

Serum FGLP1 concentrations and urine UACRs were markedly elevated in the case group compared to the healthy group (P<0.05), as shown in Table 2.

**Table 2:** Comparison of serum FGLP1 and urinary UACR between two groups (x̄±s).

Group	n	Serum FGLP1 (ng/mL)	UACR (mg/g)
Case group	256	8.36±2.22	45.32±8.47
Health Group	120	5.17±1.37	15.29±3.27
t		53.777	58.375
P		<0.01	<0.01

The serum FGLP1 concentration and urinary UACR in patients with type 2 diabetes were significantly higher than those in the healthy control group, indicating that these two indicators showed an abnormal increase trend in diabetic patients. This difference was statistically significant, suggesting that FGLP1 and UACR may be associated with the onset and progression of diabetes. The increase of FGLP1 and UACR in patients with type 2 diabetes may reflect the pathological and physiological processes of metabolic disorder and organ damage in the body. These findings provide a new perspective for understanding the mechanism of diabetic complications and suggest that FGLP1 and UACR may serve as potential biomarkers for cardiovascular risk associated with diabetes.

Univariate analysis of factors influencing the occurrence of LVDD in patients with T2DM. Compared with the non-LVDD group, the LVDD group showed statistically significant differences in age, disease duration, serum FGLP1 concentration, and UACR (P<0.05; Table 3).

**Table 3:** Univariate analysis of LVDD affecting T2DM occurrence [n (%) or x̄±s].

Item	LVDD group (n=70)	Non LVDD group (n=186)	x^2^/t	P
Age (years)			9.726	0.002
≥75	56 (80.00)	92 (49.46)		
<75	14 (20.00)	94 (50.54)		
Gender			1.213	0.274
Male	30 (42.86)	100 (53.76)		
Female	40 (57.14)	86 (46.24)		
Disease duration (years)	10.85±1.22	8.71±1.35	7.844	<0.010
Educational level			0.186	0.662
High school or vocational <br>school or below	40 (57.14)	114 (61.29)		
College or undergraduate <br>degree or above	30 (42.86)	72 (38.71)		
Body Mass Index (kg/m^2^)	20.45±1.65	20.56±1.50	0.353	0.720
Smoking history			0.510	0.475
Yes	38 (54.29)	114 (61.29)		
No	32 (45.71)	72 (38.71)		
SBP (mmHg)	155.65±2.31	155.88±2.47	0.471	0.636
DBP (mmHg)	98.52±1.47	98.64±1.31	0.075	0.946
HR (times/minute)	88.52±5.36	88.69±4.78	0.075	0.946
Serum FGLP1 (ng/mL)	9.67±1.38	7.29±1.38	8.893	<0.010
OPG (pg/mL)	72.39±3.29	73.47±4.55	1.294	0.192
SRANKL (pmol/L)	125.45±1.36	125.66±1.40	0.732	0.464
LVEF (%)	61.58±8.49	64.42±9.40	1.613	0.113
LVEDD (mm)	30.27±2.37	31.39±2.45	1.350	0.143
IVS (mm)	12.38±1.28	12.38±1.44	0.077	0.944
Cr (mmol/L)	168.41±55.41	159.47±19.40	1.373	0.176
UA (mmol/L)	337.56±68.55	332.59±70.67	0.351	0.724
TC (mmol/L)	15.38±2.20	15.28±2.47	0.214	0.837
TG (mmol/L)	13.27±2.25	13.25±1.42	0.052	0.956
LDL-C (mmol/L)	25.50±5.27	23.21±6.44	1.881	0.064
HDL-C (mmol/L)	18.59±3.28	18.69±3.45	0.142	0.884
BUN (mmol/L)	25.66±5.39	25.40±5.44	0.153	0.884
IGF-1 (ng/mL)	45.66±8.55	43.66±9.45	1.091	0.277
UACR (mg/g)	53.58±7.45	40.61±8.45	7.954	<0.010

There are significant differences in multiple clinical indicators between the left ventricular diastolic dysfunction group and the non-dysfunction group. Among them, age shows a significant difference between the two groups, suggesting that advanced age may be an important factor influencing left ventricular diastolic dysfunction. The duration of diabetes as a key indicator reflecting the severity of the disease is significantly longer in the left ventricular diastolic dysfunction group than in the non-dysfunction group, indicating that a long-term diabetic state may promote cardiac dysfunction. Serum FGLP1 concentration, a newly identified biomarker, is significantly elevated in the left ventricular diastolic dysfunction group, suggesting an association with impaired cardiac diastolic function. In addition, the urine albumin/creatinine ratio, a sensitive indicator of renal injury, shows a significant increase in the left ventricular diastolic dysfunction group, suggesting that renal dysfunction may share a common pathophysiological mechanism with cardiac diastolic dysfunction.

### Multivariate logistic regression study of risk factors for the incidence of type 2 diabetes mellitus and left ventricular diastolic dysfunction

With the occurrence of LVDD in patients as the dependent variable (occurrence = 1, non-occurrence = 0), the variables exhibiting statistically significant differences in the univariate analysis, including age, disease duration, FGLP1, and UACR, were used as independent variables for multivariate logistic analysis. Age ≥ 75 years, long disease duration, elevated UACR, and elevated serum FGLP1 concentration were independent risk factors for T2DM and LVDD (P<0.05), as shown in Table 4.

**Table 4:** Multivariate logistic analysis of LVDD occurrence in T2DM.

Influencing factors	Assignment	b	SE	Wald x^2^	P	OR	95%CI
Age ≥ 75 years old	≤ 75 years old=0; ≥ 75 years old=1	0.113	0.033	12.929	<0.01	1.119	1.054~1.188
Long course of <br>illness	Substitute raw data into	0.960	0.260	13.116	<0.01	2.622	1.551~5.212
Elevated serum <br>FGLP1	Substitute raw data into	1.091	0.286	15.078	≤0.01	2.998	1.725~1.686
Elevated UACR	Substitute raw data into	0.237	0.055	19.964	<0.01	1.266	1.143~1.403

### Relationships between serum FGLP1 concentration, urine UACR and LVDD

Spearman correlation analysis revealed that the LVDD was positively correlated with serum FGLP1 levels and the UACr (r=0.554, 0.552; both P<0.05).

This study investigated the relationships among serum FGLP1 concentration, urine UACR, and LVDD. The results showed a close correlation between them. Correlation analysis revealed that serum FGLP1 levels and UACR were positively correlated with LVDD, suggesting that these markers may be involved in the pathophysiological process of LVDD. Multivariate analysis further confirmed that elevated serum FGLP1 concentration and UACR were independent risk factors for LVDD in elderly patients with type 2 diabetes, suggesting that they may play an important role in cardiac diastolic dysfunction.

### Analysis of the predictive efficacy of serum FGLP1 levels and the UACR for LVDD

A logistic regression equation was constructed using serum FGLP1 and UACR: Y = serum FGLP1 X 1.098 + UACR X 0.234. The ROC curve was generated from the prediction model, and the area under the ROC curve (AUC) was calculated. The AUC values for serum FGLP1 concentration, UACR, and the prediction model for LVDD were 0.841, 0.899, and 0.959, respectively (see Table 5).

**Table 5:** ROC curve analysis of serum FGLP1 and UACR on LVDD.

Item	AUC	P	95%CI	Sensitivity (%)	Specificity (%)
Serum FGLP1	0.841	<0.01	0.777~0.908	80.03	77.45
UACR	0.899	<0.01	0.822~0.945	80.03	83.80
Predictive model	0.959	<0.01	0.908~0.987	91.46	88.10

## Discussion

FGLP1, a protein predominantly released by white adipose tissue, is essential for energy metabolism and glucose homeostasis. In patients with T2DM, serum levels are elevated, which may reflect a compensatory response to impaired glucose metabolism or an important marker of disease progression. An increase in the UACR reflects early kidney damage, suggesting that the kidney microvasculature in T2DM patients is already compromised and that renal filtration function is impaired, leading to increased albumin leakage [8]. Subsequent analysis indicated that age, disease duration, serum FGLP1 levels, and UACR correlated with the incidence of LVDD in T2DM patients. Furthermore, age ≥ 75 years, prolonged disease duration, increased UACR, and heightened serum FGLP1 levels emerged as independent risk factors for LVDD in this population (P<0.05). With increasing age, physiological changes in the structure and function of the heart occur, and myocardial cell compliance decreases. In addition, the prolongation of T2DM disease duration and the ongoing damage to heart muscle from high blood glucose levels increase the susceptibility of diastolic heart function. From a mechanistic perspective, a high-glucose state can trigger a series of pathological physiological changes. Persistent high glucose induces an oxidative stress response, generating large amounts of reactive oxygen species (ROS) that can damage myocardial and vascular endothelial cells. After myocardial cells are damaged, their diastolic function is affected; when vascular endothelial cells are damaged, vascular dilatory dysfunction occurs, affecting the blood supply of the heart and further aggravating myocardial damage [9][10]. Elevated glucose levels can also facilitate the formation of advanced glycation end products (AGEs). Once AGEs bind to receptors on the surface of myocardial cells, they activate intracellular signalling pathways, leading to myocardial cell hypertrophy, interstitial fibrosis, and decreased diastolic heart function. The mechanism by which serum FGLP1 is associated with LVDD may be multifaceted [11]. FGLP1 can indirectly affect cardiac function by influencing insulin sensitivity [12].

Studies have shown that FGLP1 may inhibit insulin activity, leading to increased insulin resistance and poor blood sugar control. Insulin resistance not only aggravates the disorder of sugar metabolism but also impairs glucose uptake and utilisation by cardiac muscle cells, leading to abnormal energy metabolism in heart muscle and thereby affecting diastolic function [13]. In addition, FGLP1 may participate in the development and progression of heart disease by regulating the secretion of fat factors and influencing inflammatory responses and oxidative stress. The association between elevated UACR and LVDD may stem from the close connection between the kidneys and the heart. The kidneys are important organs for regulating the body's fluid balance and excreting metabolic waste, whereas the heart is responsible for supplying the entire body with blood. When microvascular disease in the kidneys is present, and UACR increases, vascular endothelial function throughout the body may be damaged [14]. This vascular endothelial dysfunction is not limited to the kidneys but also affects heart vessels, impairing diastolic heart function. Moreover, impaired kidney function may cause water and sodium retention, increase preload, and further burden the heart, thereby promoting the occurrence and progression of LVDD [15].

The underlying mechanisms of this phenomenon are complex and involve multiple factors, including metabolic disorders, inflammatory responses, oxidative stress, and interorgan interactions. T2DM patients often have insulin resistance, which is a key factor in triggering various complications. Serum FGLP1, as a fat factor, interferes with insulin's normal function and exacerbates insulin resistance. Under conditions of insulin resistance, glucose utilisation efficiency decreases, and cardiac metabolism becomes abnormal. The energy supply to myocardial cells is insufficient, affecting their diastolic function. Moreover, insulin resistance also causes hormonal imbalance, further disrupting the normal metabolic and functional regulation of the heart and leading to the development of LVDD, thereby making LVDD positively correlated with serum FGLP1 levels. Hyperglycaemia is a characteristic of T2DM, and fluctuations in blood sugar can damage the heart and kidneys. An increase in the UACR indicates early kidney damage, often closely associated with poor glycaemic control. Long-term hyperglycaemia thickens the glomerular basement membrane, leading to increased albuminuria and higher UACR [16]. Moreover, the metabolic products generated by high blood sugar levels and their fluctuations can damage myocardial cells, reduce the compliance of the heart muscle and impair its diastolic function.

Therefore, as the UACR increases, the risk of LVDD increases as well, and the two are positively correlated. The increase in serum FGLP1 levels and the UACR is closely related to inflammatory responses. In patients with T2DM, elevated blood sugar levels stimulate the release of inflammatory cytokines. These inflammatory substances not only compromise the vascular endothelial cells of the kidneys, leading to an elevation in UACR, but also initiate cardiac inflammatory responses, impairing myocardial cell structure and function and adversely affecting diastolic function. Moreover, the inflammatory response can promote myocardial fibrosis, further aggravating LVDD, thereby establishing a positive correlation between LVDD and serum FGLP1 levels and the UACR [17][18]. Under high blood sugar conditions, a large amount of ROS is produced. When it exceeds the body's antioxidant defence capacity, ROS damage the cells and tissues of the kidneys and heart. In the kidneys, oxidative stress leads to the proliferation of glomerular mesangial cells and thickening of the basement membrane, resulting in increased UACR. In the heart, oxidative stress damages myocardial cell membranes and mitochondria, affecting energy metabolism and diastolic function [19]. An increase in serum FGLP1 may further exacerbate the oxidative stress response; through this mechanism, LVDD is positively correlated with serum FGLP1 levels and UACR.

A multivariable logistic regression equation was used to construct a predictive model including serum FGLP1 levels and UACR. The AUC of this model for predicting LVDD was 0.959, with a sensitivity of 91.46% and a specificity of 88.10%, indicating high predictive value for LVDD in patients with T2DM (P<0.05). The AUC of serum FGLP1 ranged from 0.7 to 0.9, suggesting good predictive value for LVDD. This may be because FGLP1 is a fat-related factor closely related to metabolic disorders and cardiovascular diseases. In T2DM patients, metabolic abnormalities alter FGLP1 secretion, and changes in its level can reflect cardiac function to a certain extent; thus, it can predict LVDD. However, its sensitivity and specificity did not reach very high levels, possibly because FGLP1 secretion is influenced by multiple factors, such as obesity and inflammation, which may limit its accuracy as a single indicator for diagnosing LVDD. The AUC of the UACR was relatively high, close to 0.9, suggesting good predictive accuracy for LVDD [20]. This is because the UACR reflects early kidney damage, and in T2DM patients, kidney and heart lesions are often interrelated; that is, heart-kidney syndrome. Renal microvascular lesions are associated with increased UACR but are often accompanied by changes in diastolic heart function. Therefore, the UACR can better predict the occurrence of LVDD. The sensitivity of UACR is the same as that of serum FGLP1, and UACR can detect most LVDD patients; however, a certain proportion of patients are missed.

The specificity of the UACR is slightly greater than that of serum FGLP1. This might be because the kidneys are more sensitive to changes in blood sugar. Changes in UACR can more specifically reflect pathological changes associated with T2DM and are more strongly correlated with LVDD; thus, this method performs better at excluding non-LVDD patients [21]. The area under the receiver operating characteristic (ROC) curve (AUC) of the prediction model was 0.959, indicating high diagnostic accuracy for LVDD. This is because the model integrates information from serum FGLP1 and UACR and combines it with information from different pathological processes, compensating for the limitations of a single indicator. Serum FGLP1 reflects changes in metabolism and cardiovascular aspects, whereas the UACR reflects kidney damage. The combination of the two can more comprehensively reflect the pathological state of LVDD, thereby improving the ability to predict it. The sensitivity and specificity of the prediction model are both greater than those of a single indicator, indicating that the model performs well in detecting LVDD patients and excluding non-LVDD patients. Combining multiple indicators reduces the influence of other factors on a single indicator, thereby improving diagnostic accuracy [22][23][24]. By integrating each indicator using a multivariate logistic regression equation, the model can more accurately identify LVDD patients and reduce misdiagnosis and missed diagnoses.

## Conclusion

In summary, this study demonstrated a significant correlation between serum FGLP1 levels, urinary albumin-to-creatinine ratios (UACRs), and left ventricular diastolic dysfunction (LVDD) in patients with type 2 diabetes mellitus (T2DM), elucidating the roles and mechanisms of age, disease duration, serum FGLP1 levels, and UACRs in the onset and progression of LVDD. Future research can further expand the sample size, explore in depth the interactions among these factors, and investigate whether interventions targeting them can improve cardiac function in T2DM patients, offering innovative strategies and techniques for the prevention and management of cardiovascular problems in individuals with type 2 diabetes mellitus. Moreover, clinicians should monitor serum FGLP1 levels and UACRs in T2DM patients during diagnosis and treatment to identify high-risk patients with LVDD at an early stage and implement effective interventions to improve their prognosis.

## Dodatak

### Funding

This work is supported by the National Natural Science Foundation of China (No. 82305253), National Natural Science Foundation Cultivation Project of the First Affiliated Hospital of Heilongjiang University of Traditional Chinese Medicine (PYMS202501011), Postdoctoral Science Foundation Project of Heilongjiang Province (LBH-Z24090), and China Postdoctoral Science Foundation (2025MD774056).

### Conflict of interest statement

All the authors declare that they have no conflict of interest in this work.
